# Exercise interventions for older people with cognitive frailty—a scoping review

**DOI:** 10.1186/s12877-022-03370-3

**Published:** 2022-09-01

**Authors:** Xiaohua Li, Yan Zhang, Yutong Tian, Qingyun Cheng, Yue Gao, Mengke Gao

**Affiliations:** School of Nursing and Health, Zhengzhou University, Henan, China

**Keywords:** Cognitive frailty, Older people, Exercise intervention, Scoping review

## Abstract

**Background:**

As the global population ages, the issue of frailty in older people is gaining international attention. As one of the major subtypes of frailty, cognitive frailty is a heterogeneous clinical manifestation characterised by the co-existence of physical decline and cognitive impairment. The occurrence of cognitive frailty increases the risk of adverse health outcomes in older people, affecting their daily functioning and quality of life. However, cognitive frailty is a reversible state, and many interventions have been explored, with exercise interventions playing an important role in the non-pharmacological management of cognitive frailty. This study describes and summarises current exercise interventions for older people with cognitive frailty (including parameters such as mode, frequency and duration of exercise) and identifies the limitations of existing studies to inform future exercise interventions for older people with cognitive frailty.

**Methods:**

Using a scoping review approach, Chinese and English literature published in PubMed, Web of Science, Cochrane Library, Embase, China Knowledge Network, Wanfang Database, China Biomedical Literature Database (SinoMed) and Vipshop from April 2013, when the definition of cognitive frailty first appeared, to August 2021 was searched to select studies related to exercise interventions for this group, extract information from the included literature, and summarise and report the findings.

**Results:**

Nine RCT trial studies and one quasi-experiment study were included, for a total of 10 articles. The exercise modalities involved walking, brisk walking, Otago exercise, resistance exercise, balance training, flexibility training and Baduanjin, etc.; the intensity of exercise was based on individualised guidance and graded exercise intensity; the frequency of exercise was mostly 3–4 times/week; the duration of exercise was mostly 30–60 min/time; compared to the control group, the included studies showed statistically significant improvements in cognitive function, frailty status, and depression with the exercise intervention.

**Conclusion:**

There is a paucity of evidence on exercise interventions for older people with cognitive frailty. The evidence provided in this study suggests that exercise interventions may be beneficial for older people with cognitive frailty. However, the existing studies suffer from small sample sizes, short intervention periods, inadequate monitoring of the entire exercise process, and non-uniformity in the assessment of exercise effects. More randomized controlled trials should be conducted in the future to explore the most effective, low-cost and simple interventions to meet the needs of the older people with cognitive frailty.

## Background

According to the WHO Health Statistics Report 2021, the average level of ageing (the ratio of people aged 65 and over to the total population of the region or country) has reached 9.6% worldwide, which means that approximately one in 10 is an older person [1]. The growing prevalence of an ageing population, and with this, the health of older people, is of great concern to the international community. Ageing may bring about both physical frailty and cognitive decline [2], which can occur together in an older individual. Therefore, what can be done to slow down the process of ageing and its cognitive decline is becoming a major focus of geriatric research.

In 2013, the International Academy of Nutrition and Ageing (IANA) and the International Association of Gerontology (IAGG) first proposed a definition of cognitive frailty (CF): a heterogeneous clinical manifestation characterised by the coexistence of physical frailty and cognitive impairment (Clinical Dementia Scale, CDR = 0.5) [3]. Diagnostic criteria were also specified: at least one of the frailty phenotype diagnoses (reduced body mass, fatigue, sedentary behaviour, slow gait, low muscle strength) was met, CDR = 0.5 and the exclusion of concurrent dementia or other dementias. The consensus was that cognitive frailty is characterised by a reduced cognitive reserve (which refers to the capacity of a given individual to resist cognitive impairment or decline).

In the same year, Woods AJ et al. [4] suggested that it was unreasonable to exclude patients with brain disorders from the diagnosis of cognitive frailty and concluded that reduced cognitive reserve is not a characteristic manifestation of cognitive frailty. In 2014, Dartigues JF et al. [5] disputed the issue of cognitive frailty in relation to the body, as well as the distinction between cognitive frailty and other cognitive disorders in the cognitive frailty diagnosis. In 2015, it was suggested that prefrailty should be added to the diagnostic criteria for cognitive frailty and that cognitive frailty should include two subtypes: reversible and potentially reversible [6]. In 2020, Mantovani E et al. [7] revised the definition of cognitive frailty based on a multidimensional model, whereby the assessment of cognitive frailty should include clinical features, neuropathological changes, biomarkers, disease and medication status.

As research into cognitive frailty continues, more scholars are suggesting [8–12] the use of objective biomarkers as diagnostic indicators to explore the relationship with frailty in terms of structural and functional brain performance. The definition and diagnostic criteria for CF proposed by the IANA/IAGG are widely used in current studies, and although there are differences in the choice of assessment tools for physical and cognitive function, the outcome indicators measured are consistent (i.e., physical function and cognitive function). Future research in CF will require uniform and standardised assessment tools and judgement guidelines adapted to different scenarios.

Studies have shown that the overall prevalence of cognitive frailty in older populations ranges from 0.72% to 50.10% [13], with 0.72% to 39.70% in Europe and the US and 0.76% to 50.10% in Asian populations. The onset of cognitive frailty can lead to a decline in physical function and mild cognitive impairment, resulting in a reduction in the ability to perform activities of daily living and impaired quality of life, as well as an increased risk of poor health outcomes such as malnutrition, hospitalisation, depression, incapacity, dementia and even death in older people [14].

However, cognitive frailty is reversible, and physical frailty is a dynamic process [15]. Early-stage screening and reasonable prevention and treatment measures applied in a timely manner can delay the reduction of physiological reserve capacity and reduce the occurrence of adverse health events in older people [16]. In addition, mild cognitive impairment (MCI, cognitive frailty of cognitive impairment mainly refers to mild cognitive impairment) is a transitional state between normal ageing and dementia, and MCI is reversible. It is a critical period in the management of dementia in the elderly [17]. Therefore, cognitive frailty is gaining increasing attention as a new target for healthy ageing [18] and secondary prevention of dementia [15].

Many scholars have explored interventions for cognitive frailty. Nonpharmacological interventions are currently the main modalities of cognitive frailty interventions [19], such as dietary and nutritional guidance interventions, psychosocial support, cognitive training, physical training, physiotherapy programs, etc. Exercise interventions play an important role in nonpharmacological interventions for cognitive frailty, and many scholars have conducted research on exercise interventions for elderly individuals with cognitive frailty, such as resistance exercise, individual progressive dual-task training, multicomponent exercise, and traditional Chinese medicine exercises (Baduanjin, Taijiquan), which are currently emerging. However, there is a large heterogeneity among scholars in the timing, frequency, outcome indicators, and evaluation of the effects of exercise interventions.

The aim of this study was to review the content elements, outcome types and effectiveness of exercise intervention programmes for older people with cognitive frailty, to point out problems with existing exercise intervention programmes and to provide some evidence to inform the implementation and development of future exercise intervention programmes.

## Methods

### Review approach

We used a methodologically rigorous scoping review approach to map the literature relating to exercise interventions for people with cognitive frailty in terms of the volume, methods, and characteristics of the primary research. Scoping reviews have been described as a form of comprehensive knowledge synthesis with the aim of informing practice and policy while also providing direction to research priorities [20]. An initial search of the literature revealed that there was a paucity of literature relevant to the objectives. Therefore, a scoping review approach without quantitative evaluation was used to determine what primary evidence was available.

### Establishment of the research question

To formulate the search strategy, the PCO (Population; Concept; Outcome) method was employed [21]. The population was older people with cognitive frailty, defined in accordance with the 2013 consensus of the International Academy of Nutrition and Ageing (IANA) and the International Association of Geriatrics (IAGG) that cognitive frailty (CF) is a clinical syndrome of old age characterised by cognitive impairment due to physical frailty or prefrailty, but excluding Alzheimer's disease and other dementias [3]; the concept was an exercise method was used in the intervention; the outcome indicator was the effectiveness of the exercise intervention in older individuals with cognitive frailty.

### Search strategy

Searches were conducted of PubMed, Web of Science, Cochrane Library, EMBASE, China Knowledge Network, Wanfang Database, China Biomedical Literature Database (SinoMed) and Wipu.com. A combination of subject terms and free words was used for the search. As the definition of cognitive frailty first appeared in 2013, the search time frame was from April 2013 to August 2021. The details of the search strategy were as follows: (elder^∗^ OR old^∗^ OR senior^∗^ OR geriatric^∗^) AND (“cognitive frailty” OR “cognitive decline” OR “cognitive impairment”) AND (frailty OR “frailty syndrome” OR “frailty syndrome” OR prefrail^∗^ OR frail^∗^) AND (“exercise intervention” OR “sports intervention” OR “exercise interventions” OR “movement intervention” OR “motor intervention” OR “sport intervention” OR “exercise prevention” OR “sport intervene” OR “movement therapy” OR “physical exercise”) (for example, see PubMed in Appendix A).

### Inclusion/exclusion criteria

Articles were eligible for inclusion if they were published in English or Chinese, the study population was >  = 60 years old, the study met the above definition of cognitive frailty, the study was an exercise intervention program for cognitive frailty or an integrated program with exercise interventions, and the original study had an experimental design as a randomised controlled or quasi-experimental study. Review studies, letters or conference abstracts were excluded along with literature not available in full text.

### Study selection and data extraction

The retrieved literature was imported into NoteExpress software and duplicates were screened and removed. Two researchers read the title and abstract for initial screening based on the inclusion criteria, then the full text was read for a third screening to identify the included literature, and a third researcher was asked to assist in the judgement if there was any disagreement. Data extraction included author, country, publication date, intervention target (CF Definition and Assessment Tool), intervention process, study results and so on, then summary and analysis.

## Results

**Fig. 1:**
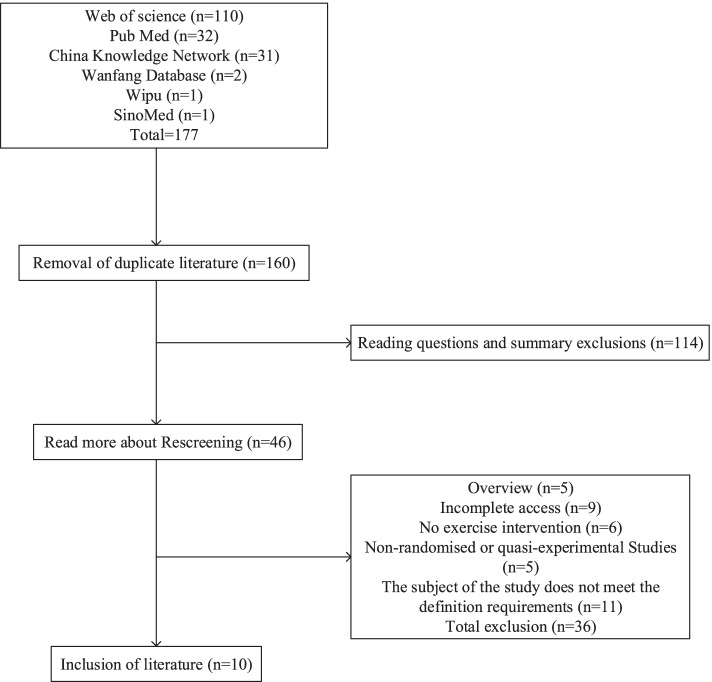
Flow chart of the literature screening

Initially, we found 177 papers. After removing duplicate papers, for the remaining 160 papers, after reading the title and abstract, 114 papers were excluded from the initial screening, and the remaining 46 papers were read in full for rescreening. A total of 36 papers were excluded, and 10 articles were ultimately included in this literature scoping review. See Fig. 1 for the literature screening process.

The included literature included nine randomised controlled studies (RCTs) [22–27, 29–31] and one quasi-experimental study [28]. The sample size of the intervention subjects ranged from 9 to 1164 cases, and the intervention duration ranged from 8 weeks to 24 months. Ten studies were conducted 1 in the USA [22], 2 in Hong Kong [23, 24], 3 in China [25–27], 1 in Singapore [28], 2 in Malaysia [29, 30] and 1 in Korea [31]. The basic information of the included literature is shown in Table 1.

**Table 1:** Basic information table of the included literature

Author	Year	Country	Types and Subjects of Research	CF definition	CF Assessment Tools	Implementer/Supervisor	Exercise interventions (content, frequency, duration, venue)	Intervention period	Personalised exercise instruction	Study results	Research limitations
Liu Z [22]	2018	USA	RCT(1164)	IANA/IAGG Standards: No cognitive frailty; prefrail without MCI; frail without MCI; nonfrail with MCI; prefrail with MCI; cognitive frailty;	Osteoporotic Fractures (SOF) index; Modified Mini-Mental State Examination (3MSE) scale	Sports coach/ unspecified	Twice weekly physical activity sessions at the Senior Activity Centre: Walking (30 min/rep), Lower body strength training (10 min/rep), Flexibility (35 min/rep), Balance; Family activities 3 to 4 times a week	24 months	Exercise intensity scale to guide exercise interventions, individualised and low to high intensity	Low odds of worsening cognitive frailty and unchanged effect of IL-6 levels on cognitive frailty at baseline	Lack of exercise process monitoring, exercise safety and effectiveness not evaluated
Kwan RY [23]	2020	Hong Kong	RCT(16/17)	Ruan Q et al. [6] Criteria: mild cognitive impairment (MCI) and physical frailty (including both frailty and prefrailty)	Clinical Dementia Rating; Montreal Cognitive Assessment (MoCA); Fried Frailty Index (FFI)	Unspecified/mobile health devices	Week 1 health education, First two weeks of brisk walking training; 3 to 12 weeks (after smartphone training) Self-paced brisk walking training + Mobile health interventions (Setting short-term personalised goals; encouraging text messages; e-coaching and reminders; self-tracking and more)	12 weeks	Electronic message alerts (personalisation); Goal grading based on baseline health, past performance, personal aspirations and so on	Improved cognitive function (in both groups);Significant reduction in frailty and improvement in walking time and stride length in the intervention group	Blindness not implemented; Small sample size; Limited applicability of the findings to people who do not walk regularly; Other confounding factors in mobile health interventions not controlled
Kwan RYC [24]	2021	Hong Kong	RCT(9/8)	IANA/IAGG Standards: The coexistence of MCI and physical frailty without being severe enough to have dementia	Montreal Cognitive Assessment (MoCA); Clinical Dementia Rating; Fried Frailty Phenotype (FFP) scale	Research Assistant	Community Service Centres: VR cognitive training and motor training. Cognitive training through simulated daily activities + video games; Physical training through cycling in a virtual environment, 2 times a week for 30 min each time	8 weeks	Adjust the amount of exercise based on participant preference and previous cycling performance; Participants and interveners consult together; Cognitive training difficulty levels	Significant improvement in cognitive function in the intervention group; Weakness was similar in both groups; Walking speed has improved	May participate in other projects at the time of intervention, confounding factors not controlled; How to train CF seniors in VR operations not explained; Sample size too small and poorly represented
Chen X [25]	2021	China	RCT(29/30)	Ruan Q et al. [6] Criteria: mild cognitive impairment (MCI) and physical frailty (including both frailty and prefrailty)	Beijing version of the Montreal Cognitive Assessment (MOCA- BJ) scale; Fried Frailty Phenotype (FFP) scale	Physiotherapist/Nurse	Health Education + 12 weeks of group OEP (Otago Campaign Program), OEP consists of 5 min of warm-up, 10 min of resistance training and 15 min of balance training;30 min per session, Monday, Wednesday and Friday interventions	3 months	Instruction according to exercise level, complete lower level tasks and move on to higher level tasks	Improved physical functional status, reduced depressive symptoms	Small sample trials with limited generalisability of results; Lack of objective data for exercise process monitoring, subjective judgement by nurses
Xia R [26]	2020	China	RCT(51/51)	Won CW’s [32] definition of CF: physical frailty; more than 1.5 standard deviations below the mean for age-, gender-, and education-adjusted norms on any cognitive function test; no dependency in instrumental activities of daily living	Edmonton Frailty Scale (EFS)-Chinese Revised; Montreal Cognitive Assessment (MoCA); Global Deterioration Scale (GDS)	Sports coach/ Intervention supervisor	Group intervention in community activity centres, health education + Ba Duan Jin training, 3 times a week for 60 min each time (including 15 min of warm-up activities, 40 min of Baduanjin exercises and 5 min of finishing activities)	24 weeks	Uniform Ba Duan Jin training, no personalised content involved	Significant improvement in frailty (moderate intensity activity, increased grip strength), improved cognitive function (total cognitive score, visuospatial, verbal and delayed memory, enhanced recall on complex graphical tests)	Lack of monitoring of the exercise process, lack of safety measures and lack of individualised programmes according to the subject's mastery of the Ba Duan Jins
Ye M [27]	2021	China	RCT(45/45)	IANA/IAGG Standards: The coexistence of MCI and physical frailty without being severe enough to have dementia	Diagnosis of MCI with reference to Petersen criteria; Fried Frailty Phenotype (FFP) scale	Rehabilitators, community doctors, family members	First 2 weeks in hospital rehabilitation unit with community practitioners and family members throughout; 3–12 weeks Nutrition promotion at CHC or home + multicomponent exercise prescription (aerobics, resistance exercise, balance training and flexibility training)Community general practitioner and family companionship and guidance, with follow-up visits every 2 weeks by a rehabilitator; 3 times a week for 45 min each time	6 months	The resistance load is set according to the patient's increased level of resistance to exercise, using the Borg Subjective Exertion Rating Scale	Useful for debilitating phenotypes, mild cognitive impairment, dietary intake, and improvement in nutritional status	How to determine if a family member has the ability to direct supervision is not indicated
Merchant RA [28]	2021	Singapore	quasi-experimental study (129)	Four CF definitions: Motoric Cognitive Risk Syndrome (MCR); Physio-cognitive Decline Syndrome (PCDS); reversible CF; potentially reversible CF	Chinese Mini-Mental State Examination (cMMSE); Montreal Cognitive Assessment (MoCA); 5-item FRAIL questionnaire	Health Coach/Unspecified	Dual task training (whole body movement exercises and cognitive training) in community activity centres: Strength training with resistance bands; Aerobic exercise using pedals and marching; Balance + subtraction/addition/naming/recall tasks; Weekly 60-min exercise sessions (20-min stretching warm-up, 40-min dual task training)	3 months	80 dual task training programmes of varying intensity, with health coaches tailoring the intensity of the workout to the participants' functional ability	Significant improvement in overall cognitive function and reduced incidence of frailty	Different types and intensities of interventions per week for the target population; More female research subjects, limited representation; Nonrandomised controlled trials
Murukesu RR [29]	2020	Malaysia	RCT (not mentioned)	IANA/IAGG Standards: The coexistence of MCI and physical frailty without being severe enough to have dementia	Fried Frailty Phenotype (FFP) scale; Clinical Dementia Rating Score	Rehabilitator/Researcher	First 12 weeks Older People's Activity Centre group intervention-Multicomponent exercise programme, includes progressive resistance training, cardio, balance and flexibility training, balance training based on OEP (Otago Exercise Program) adaptations,90 min each time. Remaining 12 weeks: family activities, 2 times a week, distribution of "WE-RISE at Home" packs (training kits)	6 months	Graded intervention goals: 1–4 weeks (level 1), 5–8 weeks (level 2), 9–12 weeks (level 3) with increasing intensity of exercise and cognitive training	Experiment in progress, no results reported	Lack of objective equipment monitoring during exercise; lack of safeguards for adherence to home interventions
Ponvel P [30]	2021	Malaysia	RCT(165/165)	IANA/IAGG Standards: The coexistence of MCI and physical frailty without being severe enough to have dementia	Mini-Mental State Examination (MMSE); Clinical Dementia Rating Scale (CDR); Fried Frailty Phenotype (FFP) scale	Sports coach/not mentioned	Activity centres: a combination of individual counselling and groups, exercise activities (multicomponent group training) 3 times a week; Remote, family guidance during outbreaks, provision of educational materials	24 month intervention + 12 month assessment for sustainability	Progressive strength training and exercise frequency, tailor-made exercise programmes based on exercise prescriptions	Experiment in progress, no results reported	Movement process monitoring not specified; baseline uniformity not guaranteed
Yoon DH [31]	2018	Korea	RCT(32/33)	IANA/IAGG Standards: The coexistence of MCI and physical frailty without being severe enough to have dementia	Mini-Mental State Examination (MMSE-K); Clinical Dementia Rating Scale (CDR); Consortium to Establish a Registry for Alzheimer’s disease; Cardiovascular Health Study (CHS) criteria	Training Instructors	High-speed resistance training at the community activity centre: elastic exercise bands, 10 min warm-up + 40 min high-speed resistance training (seated rowing, single-leg press, lateral leg raise, half-squat, etc.) + 10 min rest after exercise	4 months	The intensity of the exercise is determined by the colour of the elastic exercise band	Improved cognitive function (processing speed and executive function); improved physical function (SPPB, TUG, gait speed); improved muscle strength (grip strength, knee extension), little change in debility score	Unclear criteria for judging different exercise intensities; small sample trials with limited generalisability of results

The exercise modalities of the 10 included studies involved walking, brisk walking, Otago exercise (an evidence-based fall prevention programme for older people in the community, focusing on balance training), resistance exercise, balance training, flexibility training, and Baduanjin (a traditional Chinese qigong exercise that consists of eight movements and a safe aerobic exercise); exercise intensity included individualised instruction (*n* = 4), graded exercise intensity (*n* = 4), and uniform instruction (*n* = 2); exercise frequency tended to be 3–4 times/week (*n* = 6), with a few having 1–2 times/week (*n* = 4); and exercise duration tended to be 30–60 min/session (n = 8), with a few having 90 min/session (*n* = 1).

There was high heterogeneity of outcome measures in the 10 included studies, but no serious adverse events related to exercise were reported by any of the included studies. Compared to the control group, the included studies showed statistically significant improvements in cognitive function [23, 24, 26–28, 31], debilitating conditions [23–28, 31], depression [25], walking length and speed [23, 24, 31], grip strength [31], and nutritional status [27] after the exercise intervention, and some trials are ongoing [29, 30], with no relevant outcomes reported.

## Discussion

The aim of this study was to explore and summarise the evidence on exercise interventions relevant to older people with cognitive frailty. Most studies on cognitive frailty are epidemiological surveys and primary intervention studies, and no quantitative evaluations of exercise interventions for older people with cognitive frailty have been published. This study is the first to summarise interventions for older people with cognitive frailty. A total of 10 studies on exercise interventions for older people with cognitive frailty were included, covering a comprehensive programme of interventions, including exercise interventions. A variety of exercise intervention types exist for cognitively frail older people, such as resistance training, balance training, flexibility training and multicomponent exercise programmes, including walking, brisk walking and cycling.

The majority of the included studies showed that exercise interventions have a positive impact on older people with cognitive frailty. Among the 10 studies, six [22, 24, 27, 29–31] selected subjects for inclusion with reference to the definition of CF proposed by the 2013 IANA/IAGG group consensus, and two [23, 25] included subjects according to Ruan Q et al. [6]’s definition, taking into account the prefrailty state. Xia R et al. [26] included subjects with reference to Won CW's [32] definition, and Merchant RA et al. [28] considered four extant definitions of CF. Differences in the criteria for the inclusion of subjects and differences in screening assessment tools may have influenced the effect of the exercise intervention.

Additionally, there was a large variation in the outcome evaluation tools among the included studies, e.g., Chen X et al. [25] chose depression indicators in the outcome evaluation, and Ye M et al. [27] reported nutritional status, which was not reported in the other studies. The issues of heterogeneity in study population inclusion and uniform standardisation of outcome indicators also posed difficulties in the quantitative evaluation of exercise interventions for older people with cognitive frailty. Future studies on older people with cognitive frailty need standardised assessment tools, guidelines for different application scenarios and outcome indicators covering multiple domains, including physiological and psychological, to encourage researchers to adopt specific and authoritative assessment tools when evaluating the effects of exercise.

The continued progression of cognitive frailty can lead to a decline in the older person's ability to perform daily living activities, increasing the risk of adverse health outcomes such as hospitalisation, depression, incapacity, dementia and even death, which can seriously affect the quality of life of older people. Therefore, understanding what interventions can help delay or reverse cognitive frailty in older people may be important to reduce adverse outcomes.

Exercise interventions play an important role in nonpharmacological interventions for older people with cognitive frailty. A review of current exercise interventions also attempted to derive implications for future exercise interventions for older people with cognitive frailty: 1) for older people with CF at home in the community, exercise modalities such as walking [22] and brisk walking [23] are simple and easy to implement, while resistance training (resistance bands [28], elastic exercise bands [31] for high-speed resistance exercise and resistance exercise modalities based on multicomponent exercise [27] and Otago exercise [25]) require professional instructors and are difficult to implement at home. 2) Traditional Chinese medicine health exercises may be effective (e.g., Ba Duan Jin [26]), and the application of traditional Chinese medicine modalities such as Taijiquan [33] and educational brain health exercises in Chinese older people with cognitive frailty can be further explored in the future. 3) With the application of the mobile health concept in exercise interventions, more combined online + offline exercise interventions can be explored, such as adding mobile health supervision and guidance to traditional brisk walking training [23] and remote home exercise health guidance packages [29], giving full play to the role of wearable devices and internet technology in the field of exercise interventions [34]. 4) Exercise intensity assessment and monitoring should be uniformly standardised among exercise interventions. Existing studies have included mobile device monitoring [23], subjective judgement by the interventionist [26–29, 31], and a proportion of studies have not conducted exercise intensity monitoring [22, 30]. The American College of Sports Medicine recommends the use of heart rate combined with subjective fatigue to determine exercise intensity [35], and future exercise interventions should be a combination of subjective evaluation (exercise intensity self-rating scale [36], subjective fatigue scale [37], etc.) + objective data (heart rate, blood oxygen, blood pressure, etc.), as well as regulating the issue of supervisor qualification. 5) The effects of exercise cannot be shown in the short term, and the current studies have a short intervention duration, with eight studies having an intervention duration of less than six months [23–29, 31] and only one [30] having a 12-month continuous evaluation. The duration of intervention and follow-up should be extended in the future to clarify the long-term effects of exercise interventions on older people with cognitive frailty.

### Strengths and limitations

For this study, a scoping review methodology was chosen to map the literature related to exercise interventions for people with cognitive frailty, which can provide a reference for sports intervention in older people with cognitive frailty. No quantitative evaluation of the included studies was carried out, as this was outside the scope (or purpose) of this type of study.

## Conclusion

There is a paucity of evidence on exercise interventions for older people with cognitive frailty, and the evidence provided in this study suggests that exercise interventions may be beneficial for older people with cognitive frailty. However, more experimental studies should be conducted in the future to explore the most effective, low-cost and simple interventions to meet the needs of older people with cognitive frailty.

### Future recommendations

The results of this study show that the current research on exercise interventions for older people with cognitive frailty is small in scale and that future multicentre trials with large samples should be conducted to enhance the reliability and generalisability of the findings. A comparative study of the same type of exercise should also be conducted to explore the effects of different exercise intensities, exercise durations and frequencies on older people with cognitive frailty to clarify the optimal exercise intensity, frequency and duration.

## Statement

The method was carried out in accordance with relevant guidelines and regulations.

## Data Availability

The datasets used and/or analysed during the current study available from the corresponding author (Zhang Yan, E-mail: zhangyanmy@126.com) on reasonable request.

## Appendix A

### Search Strategy

Pubmed:

#1.(((elder*[Title/Abstract]) OR (old*[Title/Abstract])) OR (senior*[Title/Abstract])) OR (geriatric*[Title/Abstract])

#2.((("cognitive frailty"[Title/Abstract])) OR ("cognitive impairment"[Title/Abstract])) OR ("cognitive decline"[Title/Abstract])

#3.(((frailty[MeSH Terms]) OR ("frailty syndrome"[Title/Abstract])) OR (pre-frail*[Title/Abstract])) OR (frail*[Title/Abstract])

#4.(((((((((exercise intervention[Title/Abstract])) OR (sports intervention[Title/Abstract])) OR (exercise interventions[Title/Abstract])) OR (movement intervention[Title/Abstract])) OR (motor intervention[Title/Abstract])) OR (sport intervention[Title/Abstract])) OR (exercise prevention[Title/Abstract])) OR (sport intervene[Title/Abstract])) OR (movement therapy[Title/Abstract])) OR (physical exercise[Title/Abstract])

#1 and #2 and #3 and #4
